# Simplified Convolutional Neural Network Application for Cervix Type Classification via Colposcopic Images

**DOI:** 10.3390/bioengineering9060240

**Published:** 2022-05-30

**Authors:** Vitalii Pavlov, Stanislav Fyodorov, Sergey Zavjalov, Tatiana Pervunina, Igor Govorov, Eduard Komlichenko, Viktor Deynega, Veronika Artemenko

**Affiliations:** 1 Higher School of Applied Physics and Space Technologies, Peter the Great St. Petersburg Polytechnic University, 195251 St. Petersburg, Russia; fedorov_sa@spbstu.ru (S.F.); zavyalov_sv@spbstu.ru (S.Z.); 2 Personalised Medicine Centre, 197341 St. Petersburg, Russia; ptm.pervunina@yandex.ru (T.P.); govorov.igor.med@gmail.com (I.G.); e_komlichenko@mail.ru (E.K.); deynega.v.u@gmail.com (V.D.); artemenko.doc@gmail.com (V.A.)

**Keywords:** convolutional neural networks, colposcopy, pathologies, suspicious for invasion, cervical cancer

## Abstract

The inner parts of the human body are usually inspected endoscopically using special equipment. For instance, each part of the female reproductive system can be examined endoscopically (laparoscopy, hysteroscopy, and colposcopy). The primary purpose of colposcopy is the early detection of malignant lesions of the cervix. Cervical cancer (CC) is one of the most common cancers in women worldwide, especially in middle- and low-income countries. Therefore, there is a growing demand for approaches that aim to detect precancerous lesions, ideally without quality loss. Despite its high efficiency, this method has some disadvantages, including subjectivity and pronounced dependence on the operator’s experience. The objective of the current work is to propose an alternative to overcoming these limitations by utilizing the neural network approach. The classifier is trained to recognize and classify lesions. The classifier has a high recognition accuracy and a low computational complexity. The classification accuracies for the classes normal, LSIL, HSIL, and suspicious for invasion were 95.46%, 79.78%, 94.16%, and 97.09%, respectively. We argue that the proposed architecture is simpler than those discussed in other articles due to the use of the global averaging level of the pool. Therefore, the classifier can be implemented on low-power computing platforms at a reasonable cost.

## 1. Introduction

This work is devoted to the classification of cervical lesions using colposcopic images.

Cervical cancer is prevalent nowadays, especially in middle- and low-income countries. In 2020, more than half a million CC cases were registered worldwide, with 342,000 attributed deaths [1]. At the same time, CC has excellent potential for prevention on different levels, from the individual patient to the national and global levels. This is primarily due to the particular factors outlined below. First, a direct etiological factor is known—the human papillomavirus (HPV) oncogenic strains are responsible for the most neoplasms [2]. Second, there are currently vaccine prevention methods that have demonstrated their effectiveness [3]. Third, a number of countries have already embraced the benefits of preventive assessment measures to detect cervical lesions [4]. Among the latter, an important role goes to a visual assessment method of the cervical surface with the primary goal of excluding malignancy. Implementing this set of measures led several countries to dramatically reducing their incidence and mortality from CC.

At the same time, the situation with CC in Russia, unfortunately, is still far from being resolved. In 2018, more than 18,000 CC cases were registered in Russia, and more than 7500 women died from CC [1]. The HPV vaccine is still not included in the national list. The coverage of the female population with preventive examinations, including colposcopy, is recognized as insufficient [5], partly due to a lack of awareness but also primarily due to the geographical features of the country. There are many remote areas in Russia where there are no qualified specialists capable of performing and correctly interpreting colposcopic examination results.

CC might be considered the tail-end of the continuum, which covers the changes in cervical epithelium happening under the influence of HPV. The interim steps include so-called cervical intraepithelial neoplasia (CIN) of different grades. CIN may be low-grade and high-grade, with the latter having a higher potential of progressing into CC. The period under which CIN develops into CC most probably extend over several years [6]. As a representation of this fact, most high-grade lesions appear in women aged 25–35, while invasive cancer usually occurs after the age of 40 [7,8].

HPV infection is the most important independent risk factor for developing cervical precancerous lesions and, ultimately, CC. After acquiring HPV, two features play a significant role: HPV type and persistence period within the cervical epithelium. Of more than a hundred HPV types, 14 are named high-risk since they are responsible for the vast majority of advanced cervical lesions [9,10]. In most cases, HPV infection is transient, and clearance of HPV predicts CIN regression [11]. Up to 90% of new HPV infections clear out within five years [12,13]. The longer HPV stays within the cervical epithelium, the higher the risk of developing malignancy, although the factors responsible for the duration of stay are still unclear [14].

A specific part of the uterine cervix is the most susceptible to acquiring HPV infection and furthering develop dysplasia and CC. It is called the transformation zone and covers the metaplastic area, where the glandular epithelium has been replaced by the squamous. Since both de novo HPV infection and low-grade cervical lesions are often asymptomatic, specific measures must be taken to diagnose lesions before their progression to the advanced stage. In most cases, they include primary cytology screening, HPV testing, and visual assessment of the cervix during colposcopy.

The primary purpose of colposcopy is the early detection of malignant lesions of the cervix. The ultimate effectiveness of colposcopy largely depends on the operator’s experience. The interobserver variation seems to increase while inspecting the CIN1 [15]. The colposcopic examination using IFCPC criteria (2011) differentiated a healthy cervix from CIN/cancer with a specificity of 30% and a sensitivity of 86%. While distinguishing the normal cervix/CIN1 from CIN 2-3/cancer, they resulted in 94% and 61%, respectively [16]. The method’s sensitivity could be potentially increased by performing punch biopsies from multiple sites [17,18]. However, this comes with the price of increasing the invasiveness of the procedure.

Visual assessment of the state of organs was essential in managing patients with various conditions. The inner parts of the human body are usually inspected endoscopically using special equipment. For instance, each part of the female reproductive system can be examined endoscopically (laparoscopy, hysteroscopy, and colposcopy). Despite its high efficiency, this method has some disadvantages, including subjectivity and pronounced dependence on the operator’s experience.

During colposcopy, the surface of the cervix is examined with a magnifying device, which facilitates noticing the minor changes that may indicate a disease. Additional information is obtained by applying solutions of acetic acid and iodine. Based on specific patterns, the clinician concludes the need for further examination or treatment, for instance, cervical biopsy.

The main objective of this work is to develop a machine learning algorithm aimed at classifying cervical lesions. The algorithm should preferably overcome the limitations of the convenient approach, including its subjectivity, dependence on the operator experience, and demand on resources.

## 2. Related Work

There is a growing number of studies dedicated to the processing of medical images obtained during diagnostics. The main aim is to effectively diagnose diseases with decreased turnover time without losing the ultimate quality.

Prevention and early diagnostics are among the most ambitious goals of today’s medicine. It seems promising to create software for automated processing and analysis of diagnostic images, based on algorithms for computer vision and deep learning [19]. The literature provides mathematical and graphical outputs on the degree of network learning to detect pathological changes in the medical images [20]. Preliminary results published in early work show a relatively high confidence level (95%) in diagnosing cervical pathology [21,22].

A similar system for processing medical X-ray images has now been developed [23]. This system facilitates the early detection of thoracic lesions by analyzing medical images using artificial intelligence technologies. However, there is currently a lack of ready-to-use software for processing colposcopic images, to the best of our knowledge. Simultaneously, the literature review suggests that the computer vision systems trained to recognize structures show promising results.

It is essential to understand how semantic segmentation occurs in convolutional networks [22]. The University of Vietnam has developed affinity propagation clustering (APC+) to support decision-making in treating dental diseases. This method segments X-rays and selects equivalent diseases according to their classification. The most likely disease is detected using fuzzy aggregation operators. Experimental validation was performed on real dental datasets of Hanoi Medical University Hospital, Vietnam [24].

The artificial intelligence (AI) approach could also help support decision making while managing patients with acute and life-threatening conditions.

In 2007 R. Mofidi et al. [25] developed a decision support system (DSS) to classify the severity of acute pancreatitis (AP) and to predict mortality, which was based on ten clinical parameters (age, hypotension, two or more signs of systemic inflammatory response syndrome, PaO_2_, levels of lactate dehydrogenase, glucose, urea, calcium, hematocrit, and leukocyte count) measured during hospitalization and 48 h after admission to the hospital. This model performed significantly better than the commonly used APACHE II and Glasgow systems. In this paper, the sensitivity analysis was carried out to select a network’s input parameters with greater predictive informational content. The study included a relatively large number of patients with AP (n = 664): training and validation of ANNs were performed on different patients’ groups. An equally important advantage is that all ten input variables are available within the first 6 h after hospitalization. B. Andersson et al. [26] conducted a study to develop and test the effectiveness of ANN-based DSS for early prediction of the AP severity. The authors conducted a retrospective analysis of the treatment results in 208 patients with AP (from 2002 to 2005, n = 139, 2007 to 2009, n = 69). The severity of AP was determined by the criteria proposed at the conference on AP in Atlanta.

At present, the vast majority of modern medical systems are positioned by manufacturers as DSS (for example, DxPlain, IndiGO, SLIDSS, etc. [27,28]) is based on the use of statistical data analysis methods and are designed to establish a diagnosis. In surgery, the term DSS is also used to refer to preoperative planning systems. Modern preoperative planning systems allow for geometric planning, particularly for performing a complex of geometric measurements and manipulations, restoring the physiologically “normal” position of anatomical elements, and positioning and planning the choice of implants before reconstructive surgical treatment. In this regard, this article aims to identify the concept of modern decision support systems. In the DSS, based on statistical data analysis methods, they are designed to establish a diagnosis. Simultaneously, the statistical analysis of data must be carried out to structure data and patterns when working with large amounts of data that do not have an explicit structure. For this purpose, specific mathematical and algorithmic approaches can be used within the data mining field.

The images obtained during endoscopic evaluation of the gastrointestinal (GI) tract represent a valuable source for AI training. In the Norwegian Oslo University Hospital, research is being carried out on developing detection systems and assessment colorectal polyps using deep learning methods [29]. The authors were the first to use Faster R-CNN (convolutional neural network) combined with the CNN (Inception ResNet) model to detect colon polyps in images and videos. This study’s novelty lies in the post-training that can effectively reduce the number of false-positive (FP) samples. After applying several data-augmentation methods, their detection accuracy reaches 91.4%, but the average detection time is about 0.39 seconds per frame and needs further improvement. The authors used Mask R-CNN for polyp segmentation, which achieved the following results: 72.59% recall, 80% precision, 70.42% dice, and 61.24% Jaccard.

The study by Muhammad et al. [30] proposed a convolutional neural network-based approach (CNN) for classifying multiple gastrointestinal diseases using endoscopic videos that can simultaneously extract both spatial and temporal characteristics to achieve higher classification efficiency. Two different residual networks and a long-term short-term memory model are integrated into a cascade mode to extract spatial and temporal characteristics, respectively. The experimental results of the model (area under the curve 97.057%) demonstrate high performance compared with modern methods and indicate its potential for clinical use.

The article [31] proposes an automated system for detecting and classifying ulcers in wireless capsule endoscopy (WCE) images based on convolutional neural networks.

The article [32] proposes a convolutional neural network architecture of 43 convolutional layers and one fully connected layer. The network was trained and evaluated on a colonoscopy image dataset with 410 subjects provided by Gachon University Hospital. The experimental results showed an accuracy of 94.39% for 410 subjects.

Note that the colposcopic image analysis is rather complicated, mainly due to the diversity of the surface textures, their heterogeneity, and a wide range of scales. Overall, endoscopic imaging has the following features: tissue view can change significantly during underlying muscle/fiber contraction; images may have blurring; the glare effect can occur; and the brightness and contrast may vary significantly. Taken together, this requires an extensive image database embracing different conditions, as well as image defects and artefacts obtained on different hardware. Therefore, it seems reasonable to develop a system for the precise analysis of colposcopic images, which are homogeneous to a certain extent, since they are performed under clearly specified conditions, and the criteria for making a diagnosis are strictly regulated. At the same time, the task of analyzing such images has not yet been fully resolved and has not been introduced into the diagnostic practice of Russian medical institutions.

In [33], the authors used Inception-Resnet-v2 and Resnet-152 deep learning models to automatically classify cervical neoplasms in colposcopic photographs. These models are configured for two scoring systems: the cervical intraepithelial neoplasia (CIN) system and the lower anogenital squamous cell terminology (LAST) system. The multi-class classification accuracies of the networks for the CIN system in the test dataset were 48.6 ± 1.3% by Inception-Resnet-v2 and 51.7 ± 5.2% by Resnet-152. The accuracies for the LAST system were 71.8 ± 1.8% and 74.7 ± 1.8%, respectively. The area under the curve (AUC) for discriminating high-risk lesions from low-risk lesions by Resnet-152 was 0.781 ± 0.020 for the CIN system and 0.708 ± 0.024 for the LAST system.

The paper [34] proposes a model of a cervical lesion detection network (CLDNet). This network localizes and classifies the pathology in the image. The average precision of this model extraction lesion region is 92.53%, and the average recall rate is 85.56%.

There are relatively few studies in the field of classification of colposcopic images using AI [21,33,35].

Therefore, there is a growing demand for approaches that increase the population’s coverage with assessment measures, ideally without quality loss. In our opinion, these requirements are fully met by modern computer technologies, in particular, machine learning.

## 3. Methodology

**Figure 1:**
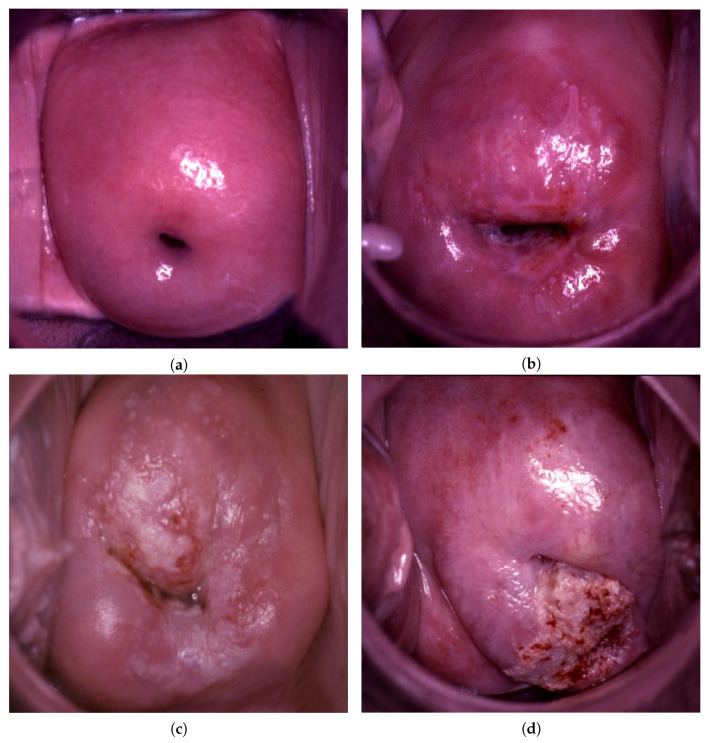
Example colposcopic images of normal cervices and various degrees of abnormal lesions: normal (**a**), LSIL (**b**), HSIL (**c**), and suspicious for invasion (**d**).

Figure 1 shows examples of colposcopic images.

First, we created a dataset. The colposcopic images were obtained from the female patients admitted to either out-patient or in-patient gynecological units of the Almazov NMRC. The images were stored in their respective local storage, connected to the colposcopes. Of note, the images were obtained retrospectively, i.e., not specifically for the current study, but rather as a part of routine examination. However, all patients were informed that the images will be stored and could potentially be used for scientific purposes, including publication. We enclosed the informed consent form with the respective point. The vast majority of patients were admitted to Almazov National Medical Research Centre, either due to suspected cervical pathology (according to previous reflex cytology and/or HPV testing) or as a part of pre-operative assessment for unrelated gynecological conditions (colposcopy is routinely performed pre-operatively, per legislation). All patients were invited to participate. The procedure was conventional and included an examination of the cervix with successive application of 5% acetic acid and 3% iodine. The collected images were de-indentified (including image metadata) and provided for further analysis. The images were pre-classified into four-classes by two experts. The experts were oncogynecologists, trained to perform colposcopy, with the first having 10 years of clinical experience and the other having 30 years of clinical experience. The possible disagreements, although rare, were solved during the consultation session with the senior specialist. The ultimate conclusion was supported by a histological examination, if available. From one to two images correspond to one patient. Therefore, there were 1500 patients in total. The images were collected and stored separately and analyzed after the removal of confidential metadata, so patient characteristics are not given, and 1500 of the 2842 images were obtained from open source data [36].

**Figure 2:**
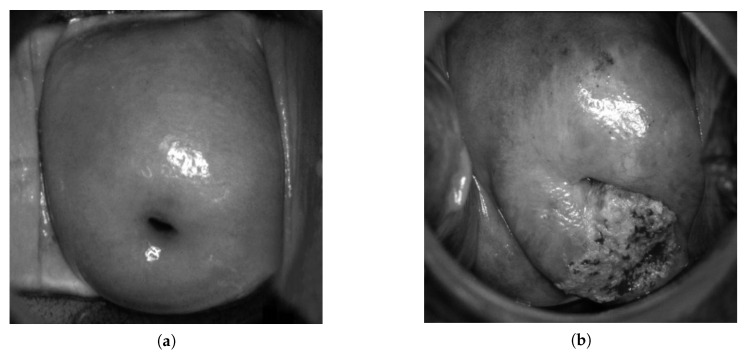
Examples of prepocessing (grayscale) of colposcopic images: normal (**a**) and suspicious for invasion (**b**).

**Figure 3:**
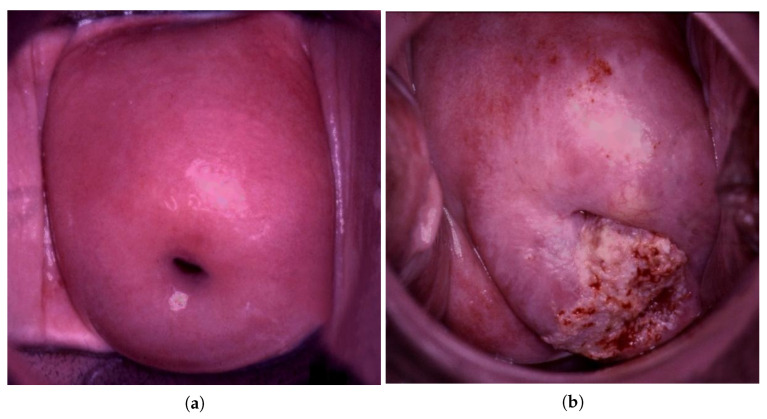
Examples of prepocessing (elimination of glares) colposcopic images: normal (**a**) and suspicious for invasion (**b**).

The initial dataset was divided into two sets for training and testing. The sample was divided according to the following principle: 70% of the sample was allocated for training and 30% for testing. Initially, the size of the training set decreased, and the size of the test set increased after each stage of training. Then, the learning process was repeated. As a result, a ratio of 1:2 images was achieved for the training and test samples (1323:657). The images were pre-classified into four classes: normal, LSIL (low-grade squamous intraepithelial lesion), HSIL (high-grade squamous intraepithelial lesion), and suspicious for invasion. Descriptions of the sets (train and test data) are presented in Table 1. To preprocess the data, we performed a grayscale conversion (Figure 2), increased resolution [37] and elimination of glare (Figure 3).

The elimination of the glare algorithm is as follows:

1. An image is converted into grayscale;
2. A grayscale image binarization is performed to select glares;
3. Each pixel in the glare is replaced with the normalized weighted sum of all known pixels in the neighborhood. This is carried out using an algorithm “An image inpainting technique based on the fast marching method” [38] that is implemented in OpenCV open source library [39].

However, during the preliminary experiments, we found that the proposed preprocessing (grayscale conversion, increased resolution, and elimination of glares) did not bring significant gains in classification accuracy.

**Figure 4:**
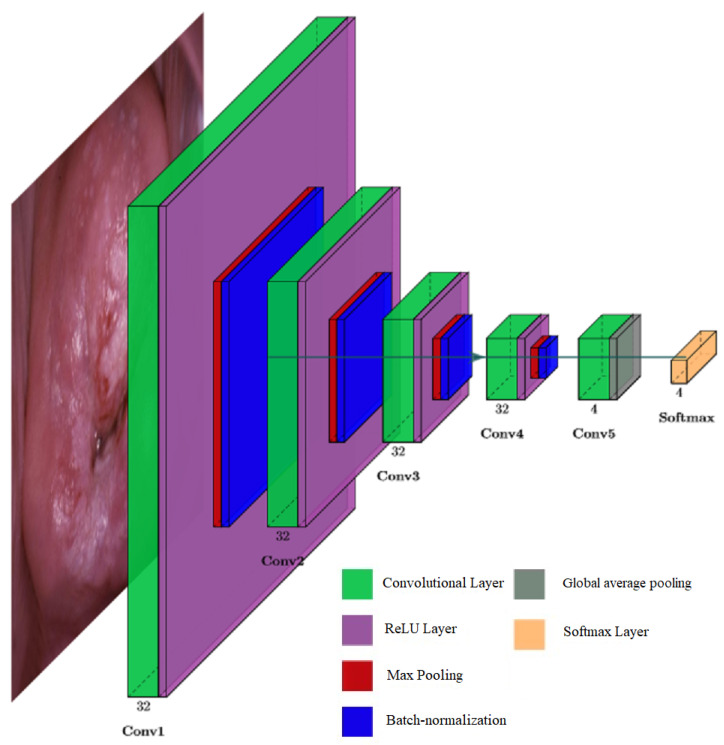
CNN Classifier architecture.

Figure 4 shows the architecture of the developed network, which has five convolutional layers (input size of 448 × 448 pixels), 32 filters with filter sizes of 3 × 3 (except the last layer), activation function ReLU, batch normalization, 4 max-pooling layers, a global average pooling layer, and a Softmax layer. Among the advantages of this network is its simple structure and, hence, low computational complexity.

Here, we provide the structure of the proposed classifier:

1. Convolutional layer is a layer that uses convolution operation for extracting features from the input.
2. ReLU is a rectified linear unit activation function. It is less prone to the vanishing gradient problem because its derivative is always 1 for the positive values of the argument.
3. Max-pooling is a subsampling layer using the maximum value. It is used to increase the receptive field.
4. Batch normalization is a method that improves performance and stabilizes the operation of neural networks. The idea of the method is that the layers of the neural network are fed with data that has been preprocessed and have zero mathematical expectation and unit variance.
5. Global average pooling is a layer that can replace a fully connected layer. As shown in [40], this layer has the same functionality as traditional fully connected layers. One of the advantages of global pooling over fully connected layers is that its structure is similar to convolutional layers, ensuring correspondence between feature maps and object categories. Additionally, unlike a fully connected layer, which requires many training and tuning parameters, reducing the spatial parameters will make the model more robust and resist overfitting.
6. Softmax is a layer for predicting the probabilities associated with a categorical distribution.

## 4. Results

Hardware systems for classification include Peter the Great Saint-Petersburg Polytechnic University Supercomputing Center and personal computer with NVIDIA GeForce GTX 2080 TI and Core i7 central processing units. First, we constructed an image dataset (Table 1), which included healthy patients and women with different cervical lesions. Unfortunately, we faced difficulties in obtaining an initially planned number of images due to the COVID-19 pandemic.

The results of the work of the classifier on the test dataset are presented in Table 2 and Table 3.

The studies were performed in accordance with recognized ethical standards. All patients signed informed consent for using the clinical images for research purposes. Additional measures were taken to remove personal data (name and date of examination) from the image metadata in order to prevent identification.

The Custom network was trained using the following parameters: Adam optimizer, categorical cross-entropy loss function, size of mini-batch is 64 (selected depending on the memory size of GPU), and 30 epochs (deviation of the loss function is not more than 5%).

**Figure 5:**
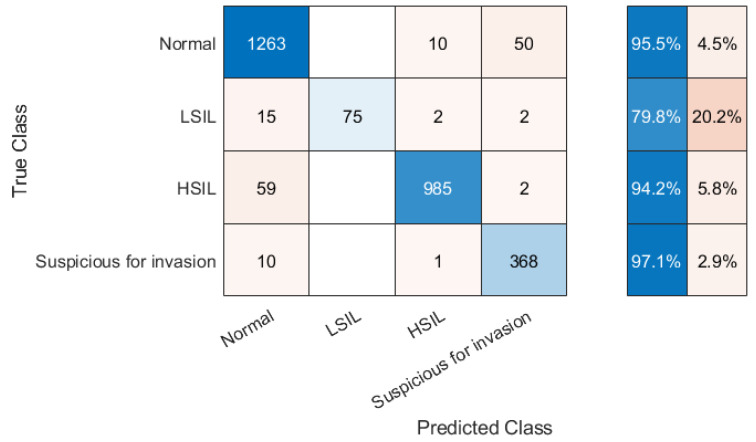
The confusion matrix for the test dataset.

The overall classification accuracy (Table 2) reached 94.68%. Figure 5 shows the confusion matrix for the test dataset. The worst result was obtained in class LSIL.

## 5. Discussion

In this paper, we describe the use of machine vision methods for the analysis of colposcopic images. Given the fact that cervical cancer is an unresolved problem in middle- and low-income countries and the obviously limited resources in these regions, the use of an automated system for primary image sorting is a reasonable alternative.

Following the purpose of the work indicated in the introduction, it is necessary to compare the obtained method with existing ones. This comparison lies not only in the plane of the obtained classification accuracy but also in the plane of computational complexity of the developed methods. Not all studies are suitable for quantitative and qualitative comparison with the results of this work. Currently, there are relatively few studies in the field of classification of colposcopic images. Moreover, there are very few studies with an assessment of computational complexity and algorithms described in detail.

This method has many advantages: it does not require repeated financial costs while being operated by the end-user. It could be implemented as software installed on computers in remote areas or regional centers. The final product has a high level of performance since the processing of one image takes approximately 20 ms. Simultaneously, technology is continuously being improved and becomes better as new images are uploaded.

Among the advantages of the method should be noted the simple structure and low computational complexity (around $39\times 10^{6}$ floating-point operations), which allows for using low-power and low-cost devices. The Nvidia Jetson family is suitable for this task due to the presence of graphics accelerator. In addition to Nvidia solutions, other embedded systems can be used, on the basis of which medical tools such as colposcopes can be created. The requirements of low computational complexity and simplicity of the structure are preserved. For comparison, the approaches from the article [21] have computational complexity equal to around $193\times 10^{6}$ (despite the fact that the network consists of only 3 convolutional layers, a fully connected layer will contain a lot of computational operations).

In a related work [14], a relatively small accuracy in validation was noted—only 50%. This indicates poor learning ability of the network. Due to the proposed approach, a number of advantages were achieved:

– The training set is two times smaller than the test set, which has a positive effect in conditions of a shortage of colposcopic images in the field of cervical cancer;
– The neural network showed good learnability with the maximum achieved classification accuracy of 94.68% on the tested data set;
– The computational complexity is 5 times less than that of existing solutions [14]. This is especially important since the placement of neural networks in medical tools, such as a colposcope, imposes performance requirements with limited resources of the computer system embedded in the tool.

**Figure 6:**
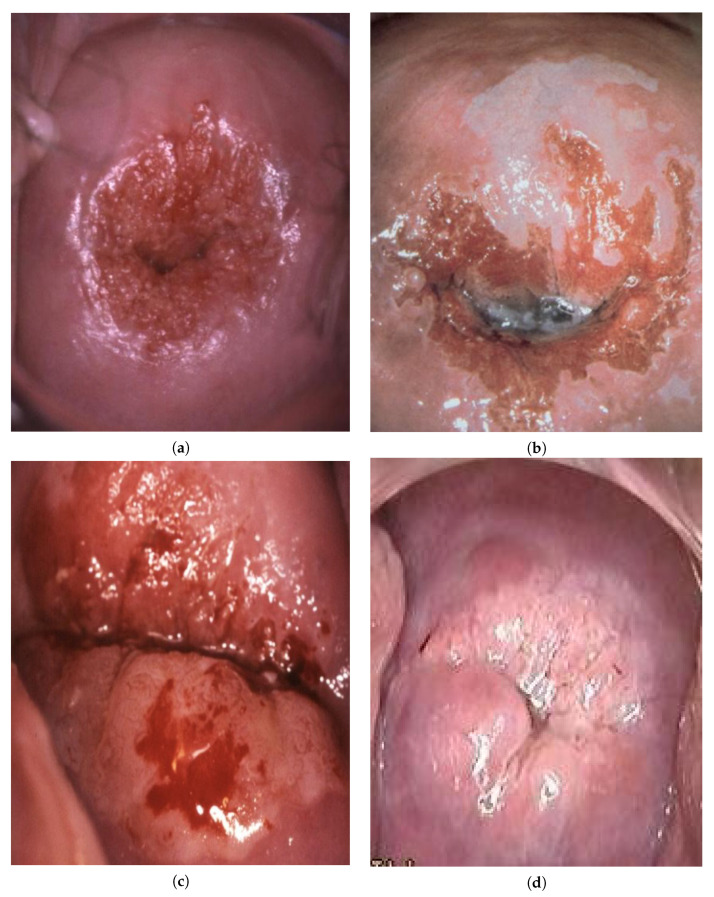
Examples of errors in decisions (predicted/real): (**a**) suspicious for invasion/normal, (**b**) suspicious for invasion/HSIL, (**c**) suspicious for invasion/HSIL, and (**d**) LSIL/HSIL.

The main task today is to increase the accuracy of the system by reducing the number of errors. Below, we show examples of the misclassification, that, although rare, represent the field for further improvement (Figure 6).

In the first case (Figure 6a), the prediction was suspicious for invasion and the real classification was normal. The roughness in the central area can be somewhat confusing, and the decision was made in favor of suspicious for invasion. From an experienced doctor’s point of view, there is additional information: its location around the external os and non-interruptured borders most likely indicate a simple case of the “columnar epithelium”, which means that this case is “normal”.

In two other examples (Figure 6b,c), the prediction was suspicious for invasion and the real classification was HSIL. Although there were no apparent signs of invasion, an advanced lesion was evident, requiring additional measures (e.g., biopsy). Therefore, although being overestimated, the predicted result does not negatively affect clinical decisions but instead increases alertness.

In one more example (Figure 6d), the prediction was LSIL and the real classification was HSIL. Here, one may note that dense acetowhite lesions are located within thin and opaque acetowhite areas, favoring more advanced lesions. However, there were no signs of invasion.

Of note, diagnosing LSIL also has the higher interobserver variability from assessing clinicians. This is because LSIL has an appearance that sometimes overlaps with the healthy cervix. In our work, this could also be because of the insufficient number of samples within the group.

Therefore, the abovementioned cases are challenging and may require additional clinical measures. When implementing the algorithm in the hardware, we plan to call alert messages for further actions (additional analyses or verification on a council of experienced doctors). Such parsed results must be taken into account in the next training iteration.

Among the possible limitations of the works, one should note that images were initially classified manually. Even though two experienced clinicians assessed the images independently, with several of the assessments, experts acknowledged the lack of consensus. In this case, additional measures were taken to reduce possible errors, such as counseling by a senior specialist. However, some minor inaccuracies may still be present.

**Figure 7:**
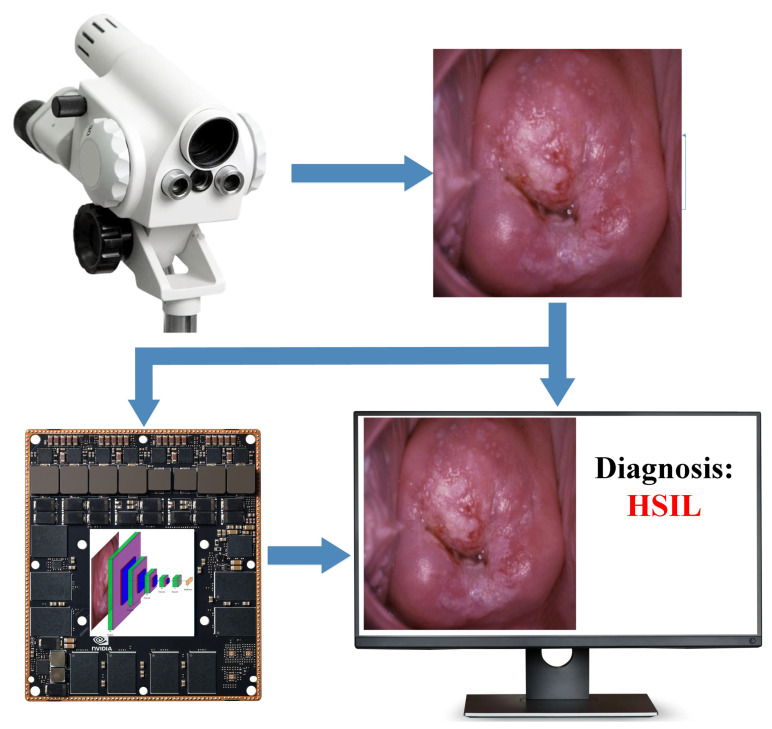
Illustration of the application of the developed approach.

The proposed approach can be used as an auxiliary diagnosis system, as shown in Figure 7. In this case, the colposcope is connected to the device based on Nvidia Jetson via HDMI cable (for example, colposcope “ZEISS OPMI pico colpo”, which we used in the Almazov National Medical Research Centre, provided this functionality). This standalone smart device with a user-friendly interface contains a CNN developed and implemented in PyTorch C++. It does not require a mobile or desktop computer connection. A monitor, touch screen, mouse, or keyboard can be used to work with it without requiring technical qualifications. It is important to note that the system does not complicate the routine procedure for the operator; does not require additional technical skills; and performs and analysis in the background, providing a result upon request.

This type of system can be improved to a smart medical autonomous distributed system for diagnostics based on machine leaning technology [41]. This concept presupposes the construction of several data centers. Data centers can collect impersonal information from doctors about some cases of misclassification. Such examples are then used to update CNN. Updated firmware for the Nvidia Jetson module can be transferred via the 5G/6G communication channel (a description of 5G/6G can be found in [42,43]). Additionally, arguable cases can be handled by creating a distributed council of experienced doctors connected to data centers to process complex colonoscopy images.

Combining of a digital colposcope, mobile computing platform and deep learning will create a “smart colposcope”. It can be part of the Internet of Medical Things (IoMT).

Many researchers note the pragmatism of using artificial intelligence in processing large amounts of data. Taking into account the number of colposcopic assessments performed worldwide, the AI approach might potentially contribute to improvements within the field. These requirements are fulfilled by modern computer technologies, in particular machine learning. Taking into account the improvement of the performance indicators of the machine vision system as new information becomes available, we would like to welcome joint efforts around the world. This approach is entirely consistent with the strategy for reducing the incidence of cervical cancer proposed b they WHO.

Of course, this is not about excluding the participation of a certified doctor in the diagnosis of cervical diseases. In its current form, this does not seem very easy to implement from technological and legal points of view. At the same time, the use of such a system as a decision support tool looks feasible, especially in countries with insufficient resources, where decreasing the incidence rate of cervical cancer is particularly important. Recently, several approaches have been proposed to eliminate the need to use expensive colposcopes (for instance, [44]). Such an approach could be implemented for use by midwives.

The strength of the proposed method its relatively low computational complexity and simple architecture. Its weakness can be attributed to the still significant amount of data set required for training. However, such a drawback is typical for all solutions based on learning.

Further research will focus on segmentation to localize and visualize pathologies on images. In medical imaging, these segments are usually commensurate with different tissues, pathologies, organs, or other biological structure. The following architectures can be distinguished: FCN [45], Deconvolution network [46], SegNet [47], U-Net [48], PSPNet [49], DeepLab [50], Mask-R-CNN [51], EncNet [52], and YOLACT [53].

## 6. Conclusions

The current article covers the experience of using a convolutional neural network to classify cervical lesions based on colposcopic images. Currently, the classification accuracy reached 94.68%. The proposed architecture is simpler than those discussed in other articles due to the application of a global averaging pooling layer. Due to its simple structure, the classifier can be implemented on low-power computing platforms at a reasonable price. Of interest, it could potentially be utilized to process images from both a wide range of machines (endoscope, X-Ray, and MRI) and handheld devices, such as dermatoscope or even smartphones, which is also proposed in other works. This will allow for diversifying and speeding up the analysis.

Machine vision is a valuable method that could be applied to analyzing medical images, including colposcopic. Such an approach becomes even more important in countries where universal cervical assessment is not yet established and/or in remote areas. The challenging next step is to improve the sensitivity and specificity of the method by enlarging the training set. Additionally, due to the low architectural complexity of the neural network in the proposed approach and due to its low computational complexity, it becomes possible to implement it for use in medical instruments such as a smart colposcope.

## Figures and Tables

**Table 1:** Description of the dataset.

*Data Type*	*Normal*	*LSIL*	*HSIL*	*Suspicious for Invasion*
*Train*	657	63	133	38
*Test*	1323	94	1046	379

**Table 2:** Accuracy on the test dataset.

*Normal*	*LSIL*	*HSIL*	*Suspicious for Invasion*	*All*
95.46%	79.78%	94.16%	97.09%	94.68%

**Table 3:** Comparison of Methods.

*Method*	*Accuracy*	*Computational Complexity*
Our	94.68%	$39\times 10^{6}$
[21]	≈50%	$193\times 10^{6}$
[35]	86%	-

## Abbreviations

The following abbreviations are used in this manuscript:

AI	Artificial Intelligence
ANN	Artificial Neural Network
AUC	Area Under the Curve
APC	Affinity Propagation Clustering
CC	Cervical Cancer
CIN	Cervical Intraepithelial Neoplasia
CNN	Convolutional Neural Network
DSS	Decision Support System
HPV	Human Papillomavirus
HSIL	High-Grade Squamous Intraepithelial Lesion
IOMT	Internet of Medical Things
LAST	Lower Anogenital Squamous Cell Terminology
LSIL	Low-Grade Squamous Intraepithelial Lesion
WCE	Wireless Capsule Endoscopy
WHO	World Health Organization

## References

[1] Sung H., Ferlay J., Siegel R., Laversanne M., Soerjomataram I., Jemal A., Bray F. (2021). Global cancer statistics 2020: GLOBOCAN estimates of incidence and mortality worldwide for 36 cancers in 185 countries. CA Cancer J. Clin..

[2] Franco E., Schlecht N., Saslow D. (2003). The epidemiology of cervical cancer. Cancer J..

[3] Lei J., Ploner A., Elfström K., Wang J., Roth A., Fang F., Sundström K., Dillner J., Sparén P. (2020). HPV vaccination and the risk of invasive cervical cancer. N. Engl. J. Med..

[4] Simms K., Steinberg J., Caruana M., Smith M., Lew J., Soerjomataram I., Castle P., Bray F., Canfell K. (2019). Impact of scaled up human papillomavirus vaccination and cervical screening and the potential for global elimination of cervical cancer in 181 countries, 2020–99: A modelling study. Lancet Oncol..

[5] Barchuk A., Bespalov A., Huhtala H., Chimed T., Laricheva I., Belyaev A., Bray F., Anttila A., Auvinen A. (2018). Breast and cervical cancer incidence and mortality trends in Russia 1980–2013. Cancer Epidemiol..

[6] Goldie S., Grima D., Kohli M., Wright T., Weinstein M., Franco E. (2003). A comprehensive natural history model of HPV infection and cervical cancer to estimate the clinical impact of a prophylactic HPV-16/18 vaccine. Int. J. Cancer.

[7] World Health Organization (2009). Human papillomavirus vaccines: WHO position paper = Vaccins anti-papillomavirus humain: Note d’information de l’OMS. Wkly. Epidemiol. Rec..

[8] Finnish Cancer Registry Cancer Statistics Site. https://syoparekisteri.fi/tilastot/tautitilastot/.

[9] Demarco M., Egemen D., Raine-Bennett T., Cheung L., Befano B., Poitras N., Lorey T., Chen X., Gage J., Castle P. (2020). A Study of Partial Human Papillomavirus Genotyping in Support of the 2019 ASCCP Risk-Based Management Consensus Guidelines. J. Low. Genit. Tract Dis..

[10] Bosch X., Harper D. (2006). Prevention strategies of cervical cancer in the HPV vaccine era. Gynecol. Oncol..

[11] Nobbenhuis M., Helmerhorst T., van den Brule A., Rozendaal L., Voorhorst F., Bezemer P., Verheijen R., Meijer C. (2001). Cytological regression and clearance of high-risk human papillomavirus in women with an abnormal cervical smear. Lancet.

[12] Ho G., Bierman R., Beardsley L., Chang C., Burk R. (1998). Natural history of cervicovaginal papillomavirus infection in young women. N. Engl. J. Med..

[13] Moore E., Danielewski J., Garland S., Tan J., Quinn M., Stevens M., Tabrizi S. (2011). Clearance of Human Papillomavirus in Women Treated for Cervical Dysplasia. Obstet. Gynecol..

[14] Rodríguez A., Schiffman M., Herrero R., Wacholder S., Hildesheim A., Castle P., Solomon D., Burk R., Proyecto Epidemiológico Guanacaste Group (2008). Rapid clearance of human papillomavirus and implications for clinical focus on persistent infections. J. Natl. Cancer Inst..

[15] Ferris D., Litaker M. (2008). Interobserver Agreement for Colposcopy Quality Control Using Digitized Colposcopic Images During the ALTS Trial. J. Low. Genit. Tract Dis..

[16] Bornstein J., Bentley J., Bösze P., Girardi F., Haefner H., Menton M., Perrotta M., Prendiville W., Russell P., Sideri M. (2012). Colposcopic terminology of the International Federation for Cervical Pathology and Colposcopy. Obstet. Gynecol..

[17] Gage J., Hanson V., Abbey K., Dippery S., Gardner S., Kubota J., Schiffman M., Solomon D., Jeronimo J. (2006). ASCUS LSIL Triage Study (ALTS) Group. Number of cervical biopsies and sensitivity of colposcopy. Obstet. Gynecol..

[18] Pretorius R.G., Zhang W.H., Belinson J.L., Huang M.N., Wu L.Y., Zhang X., Qiao Y.L. (2004). Colposcopically directed biopsy, random cervical biopsy, and endocervical curettage in the diagnosis of cervical intraepithelial neoplasia II or worse. Obstet. Gynecol..

[19] Topol E. (2019). High-performance medicine: The convergence of human and artificial intelligence. Nat. Med..

[20] Jha S., Topol E.J. (2016). Adapting to Artificial Intelligence: Radiologists and Pathologists as Information Specialists. JAMA.

[21] Sato M., Horie K., Hara A., Miyamoto Y., Kurihara K., Tomio K., Yokota H. (2018). Application of deep learning to the classification of images from colposcopy. Oncol. Lett..

[22] Fernandes K., Cardoso J., Fernandes J. (2018). Automated Methods for the Decision Support of Cervical Cancer Screening Using Digital Colposcopies. IEEE Access.

[23] AI-Powered Radiology Platform. https://botkin.ai/.

[24] Ngan T., Tuan T., Son L., Minh N., Dey N. (2016). Decision making based on fuzzy aggregation operators for medical diagnosis from dental X-ray images. J. Med. Syst..

[25] Mofidi R., Duff M., Madhavan K., Garden O., Parks R. (2007). Identification of severe acute pancreatitis using an artificial neural network. Surgery.

[26] Andersson B., Andersson R., Ohlsson M., Nilsson J. (2011). Prediction of severe acute pancreatitis at admission to hospital using artificial neural networks. Pancreatology.

[27] Chen W., Cockrell C., Ward K., Najarian K. Intracranial Pressure Level Prediction in Traumatic Brain Injury by Extracting Features from Multiple Sources and Using Machine Learning Methods. Proceedings of the IEEE International Conference on Bioinformatics and Biomedicine (BIBM ’10).

[28] Davuluri P., Wu J., Ward K., Cockrell C., Najarian K., Hobson R. An Automated Method for Hemorrhage Detection in Traumatic Pelvic Injuries. Proceedings of the 2011 Annual International Conference of the IEEE Engineering in Medicine and Biology Society.

[29] Shin Y., Qadir H., Aabakken L., Bergsland J., Balasingham I. (2018). Automatic Colon Polyp Detection Using Region Based Deep CNN and Post Learning Approaches. IEEE Access.

[30] Owais M., Arsalan M., Choi J., Mahmood T., Park K. (2019). Artificial intelligence-based classification of multiple gastrointestinal diseases using endoscopy videos for clinical diagnosis. Clin. Med..

[31] Alaskar H., Hussain A., Al-Aseem N., Liatsis P., Al-Jumeily D. (2019). Application of Convolutional Neural Networks for Automated Ulcer Detection in Wireless Capsule Endoscopy Images. Sensors.

[32] Park H., Kim Y., Lee S. (2020). Adenocarcinoma Recognition in Endoscopy Images Using Optimized Convolutional Neural Networks. Appl. Sci..

[33] Cho B.J., Choi Y.J., Lee M.J., Kim J.H., Son G.H., Park S.H., Kim H.B., Joo Y.J., Cho H.Y., Kyung M.S. (2020). Classification of cervical neoplasms on colposcopic photography using deep learning. Sci. Rep..

[34] Bing B., Yongzhao D., Peizhong L., Pengming S., Ping L., Yuchun L. (2020). Detection of cervical lesion region from colposcopic images based on feature reselection. Biomed. Signal Process. Control.

[35] Xue P., Tang C., Li Q., Li Y., Shen Y., Zhao Y., Chen J., Wu J., Li L., Wang W. (2020). Development and validation of an artificial intelligence system for grading colposcopic impressions and guiding biopsies. BMC Med..

[36] Kaggle Intel & MobileODT Cervical Cancer Screening. https://www.kaggle.com/c/intel-mobileodt-cervical-cancer-screening.

[37] Kim J., Lee J., Lee K. Accurate Image Super-Resolution Using Very Deep Convolutional Networks. Proceedings of the IEEE Conference on Computer Vision and Pattern Recognition (CVPR).

[38] Telea A. (2004). An Image Inpainting Technique Based on the Fast Marching Method. J. Graph. Tools.

[39] OpenCV Site. https://opencv.org/.

[40] Lin M., Chen Q., Yan S. (2013). Network In Network. arXiv.

[41] Velichko E., Nepomnyashchaya E., Baranov M., Galeeva M., Pavlov V., Zavjalov S., Savchenko E., Pervunina T., Govorov I., Komlichenko E. (2019). Concept of Smart Medical Autonomous Distributed System for Diagnostics Based on Machine Learning Technology. Lecture Notes in Computer Science (Including Subseries Lecture Notes in Artificial Intelligence and Lecture Notes in Bioinformatics).

[42] Tataria H., Shafi M., Molisch A., Dohler M., Sjöland H., Tufvesson F. (2021). 6G Wireless Systems: Vision, Requirements, Challenges, Insights, and Opportunities. Proc. IEEE.

[43] Akyildiz I., Kak A., Nie S. (2020). 6G and Beyond: The Future of Wireless Communications Systems. IEEE Access.

[44] MobileODT Site. https://www.mobileodt.com/.

[45] Long J., Shelhamer E., Darrell T. Fully convolutional networks for semantic segmentation. Proceedings of the IEEE Conference on Computer Vision and Pattern Recognition (CVPR).

[46] Noh H., Hong S., Han B. Learning deconvolution network for semantic segmentation. Proceedings of the IEEE International Conference on Computer Vision (ICCV).

[47] Badrinarayanan V., Kendall A., Cipolla R. (2017). SegNet: A Deep Convolutional Encoder-Decoder Architecture for Image Segmentation. IEEE Trans. Pattern Anal. Mach. Intell..

[48] Ronneberger O., Fischer P., Brox T. (2015). U-Net: Convolutional Networks for Biomedical Image Segmentation. Medical Image Computing and Computer-Assisted Intervention—MICCAI.

[49] Zhao H., Shi J., Qi X., Wang X., Jia J. Pyramid scene parsing network. Proceedings of the IEEE Conference on Computer Vision and Pattern Recognition (CVPR).

[50] Chen L., Papandreou G., Kokkinos I., Murphy K., Yuille A. (2018). DeepLab: Semantic Image Segmentation with Deep Convolutional Nets, Atrous Convolution, and Fully Connected CRFs. IEEE Trans. Pattern Anal. Mach. Intell..

[51] He K., Gkioxari G., Dollar P., Girshick R. Mask R-CNN. Proceedings of the IEEE International Conference on Computer Vision (ICCV).

[52] Zhang H., Dana K., Shi J., Zhang Z., Wang X., Tyagi A., Agrawal A. Context Encoding for Semantic Segmentation. Proceedings of the IEEE/CVF Conference on Computer Vision and Pattern Recognition.

[53] Bolya D., Zhou C., Xiao F., Lee Y. YOLACT: Real-Time Instance Segmentation. Proceedings of the IEEE/CVF International Conference on Computer Vision (ICCV).

